# *Mycobacterium ulcerans* in Possum Feces before Emergence in Humans, Australia

**DOI:** 10.3201/eid3103.240657

**Published:** 2025-03

**Authors:** Bridgette J. McNamara, Jack Cornish, Kim R. Blasdell, Eugene Athan, Naomi E. Clarke, Tiffany Pe, Mohammad Akhtar Hussain, Michael Muleme, Ee Laine Tay, Michael Dunn, Victoria Boyd, Anjana Karawita, Daniel P. O’Brien

**Affiliations:** Barwon Health, Geelong, Victoria, Australia (B.J. McNamara, J. Cornish, E. Athan, N.E. Clarke, T. Pe, M.A. Hussain, M. Muleme, D.P. O'Brien); Centre for Innovation in Infectious Disease and Immunology Research, Geelong (B.J. McNamara, E. Athan, M.A. Hussain, M. Muleme, D.P. O'Brien); The University of Melbourne, Melbourne, Victoria, Australia (B.J. McNamara, D.P. O'Brien); CSIRO Australian Centre for Disease Preparedness, Geelong (K.R. Blasdell, M. Dunn, V. Boyd, A. Karawita); Deakin University, Geelong (E. Athan, T. Pe, M.A. Hussain); Victoria Department of Health, Melbourne (E.L. Tay)

**Keywords:** *Keywords:* Buruli ulcer, Mycobacterium ulcerans, tuberculosis and other mycobacteria, bacteria, zoonoses, epidemiology, incidence, possum reservoir hosts, surveillance, urban outbreak, prevention, Australia

## Abstract

We describe emergence of Buruli ulcer in urban Geelong, Victoria, Australia, and examine timing and proximity of human cases to detection of *Mycobacterium ulcerans* DNA in possum feces. *M. ulcerans*–positive feces preceded human cases by up to 39 months, constituting an early warning of impending risk for Buruli ulcer.

Buruli ulcer is a neglected tropical disease and remains a public health issue across south-eastern Australia (*1*). Manifesting as a necrotizing ulcer, Buruli ulcer is caused by the environmental pathogen *Mycobacterium ulcerans* (*2*). Although global case numbers are falling, the ongoing epidemic in Victoria, Australia, appears to be worsening (*3*), with a record 362 notified cases in Victoria in 2023. Historically concentrated in coastal areas in Victoria, more recent cases have emerged within noncoastal urban suburbs of Melbourne and Geelong (*4*); the reasons remain unclear. Temporal analysis of the phylogenic trees of Victoria *M. ulcerans* isolates suggests a 7- to 9-year lag time between the bacterium’s arrival in an area and the emergence of human cases (*5*). Human acquisition routes may include mosquito bites (*6*), direct trauma, or contact with contaminated substrates, particularly in limb areas with exposed skin (*2*,*7*). Unlike in Africa, Australia shows evidence of zoonotic transmission, notably from native ringtail (*Pseudocheirus peregrinus*) and brushtail (*Trichosurus vulpecula*) possums, which inhabit native habitat across coastal and inland areas of Australia (*8*). Possum feces testing positive for *M. ulcerans* DNA found at residential properties correlated with higher likelihood of Buruli ulcer among residents (*9*); surveillance in possums enhances case prediction in humans (*10*), yet the timing of infection in possum populations related to the emergence of human cases has not been described. We describe the emergence of human Buruli ulcer cases in Geelong, Victoria, Australia, and their timing and proximity to *M. ulcerans*–positive possum feces.

## The Study

**Figure 1:**
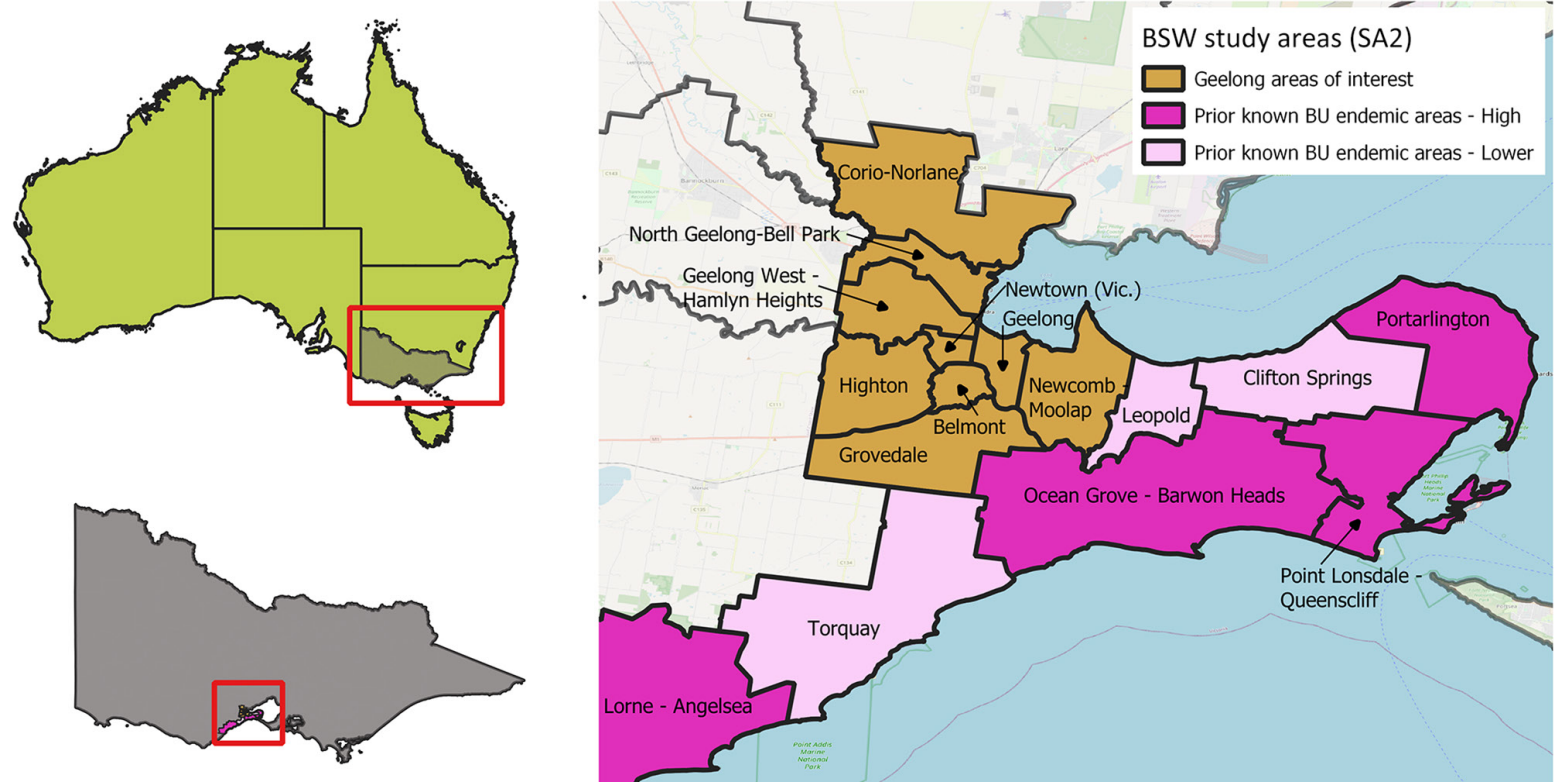
Location of central Geelong suburbs in Victoria, Australia, and prior known endemic areas in the Bellarine Peninsula and Surf Coast (higher case numbers dark pink, lower case numbers in light pink). We defined high incidence as >10 cases/100,000 population in 2017–2019. Belmont with the central Geelong suburbs also meets this criterion. SA2, Australian Bureau of Statistics Statistical Area 2.

We conducted a descriptive epidemiologic study, including a spatiotemporal clustering analysis of laboratory-confirmed and probable Buruli ulcer cases notified to the Department of Health Victoria during January 1, 2011–December 31, 2022, to identify new endemic areas in suburbs of Geelong, a city of ≈250,000 persons in southwest Victoria, Australia (Figure 1). We examined aggregate case numbers and incidence per 100,000 estimated resident population (*11*) at Australian Bureau of Statistics (ABS) Statistical Areas 2 (SA2) for the central Geelong suburbs and known endemic areas of the Bellarine Peninsula and Surf Coast. We examined geographic clustering of Geelong cases, mapped to ABS Meshblocks (small geographic areas, 3–40 dwellings), in a space-time analysis using Poisson models in SatScan (http://www.satscan.org). The models scan across geographic locations over time to detect areas and time periods where reported case numbers significantly exceed expected case numbers.

We used data from a systematic possum fecal survey conducted in Geelong suburbs in 2020 (*10*), as well as follow-up surveys conducted in a more restricted area of Geelong at 3, 6, and 24 months later and several samples from Belmont in the presurvey period in September 2019 (Appendix Figure 1). Fecal survey collection methods have been published previously (*10*,*12*). We used the data to examine the proximity and timing of human Buruli ulcer cases notified in 2020–2022 to feces from *M. ulcerans*–infected possums by calculating distance from Buruli ulcer case residence (in meters) and timing (in months) from the most proximal possum fecal sample collection to human case at notification.

**Figure 2:**
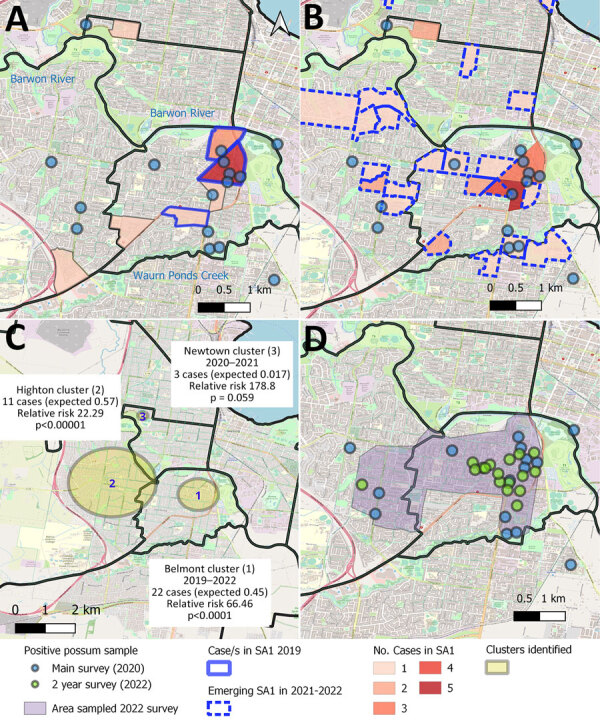
Distribution, clustering, and timing of Buruli ulcer cases in relation to *Mycobacterium ulcerans*–positive possum fecal samples, Victoria, Australia. A) Number and home location of human Buruli ulcer cases in Statistical Area SA1 in 2019–2020, compared with the distribution of *M. ulcerans*–positive possum fecal samples collected in the systematic main survey in 2020. Solid blue outline indicates areas in 2019 in which cases were tightly clustered during 2019; colored areas without borders had cases in 2020 only, B) Number and home location of human Buruli ulcer cases in Statistical Area SA1 in 2021–2022, compared with the distribution of *M. ulcerans*–positive possum fecal samples collected in the systematic main survey in 2020. Dashed blue outline indicates areas with cases only in 2021–2022 and not 2019–2020. Blue circles indicate 100-meter radius around the collection location. C) Spatiotemporal clustering of human Buruli ulcer cases from 2011–2022 in Geelong suburbs, Australia. The observed number of cases within each cluster are compared to the expected number for the estimated resident population of the area during the period (*3*), under the null hypothesis (no spatiotemporal clustering). D) Changing distribution of positive possum fecal samples from 2020 (blue) and 2022 (green).

During 2011–2022, a total of 402 Buruli ulcer cases were reported in the Barwon South West (BSW) catchment, including 80 cases within the SA2 locations of interest in central Geelong. We observed increasing case numbers and incidence in Belmont during 2017–2022 and in Highton and Newtown areas during 2020–2022 (Table 1); we identified 3 clusters where reported case numbers exceeded the number expected (Figure 2, panel C).

**Table 1:** Cases and incidence of Buruli ulcer in a noncoastal urban area, Australia, 2011–2020*

Location	2022 population†	No. cases (no. per 100,000 patient-years)			
		2011–2013	2014–2016	2017–2019	2020–2022
Geelong SA2					
Belmont	14,829	3 (7.2)	0 (0.0)	9 (**20.5**)	24 (**53.5**)
Corio–Norlane	27,622	0 (0.0)	0 (0.0)	2 (2.4)	0 (0.0)
Geelong	13,781	2 (5.3)	0 (0.0)	1 (2.5)	1 (2.4)
Geelong West–Hamlyn Heights	21,272	2 (3.4)	0 (0.0)	0 (0.0)	2 (3.1)
Grovedale	31,579	0 (0.0)	0 (0.0)	3 (3.6)	4 (4.0)
Highton	23,869	3 (4.9)	1 (1.5)	1 (1.4)	12 (**16.6**)
Newcomb–Moolap	15,089	3 (6.7)	0 (0.0)	0 (0.0)	0 (0.0)
Newtown (Victoria.)	10,945	0 (0.0)	0 (0.0)	1 (3.1)	6 (**18.1**)
North Geelong–Bell Park	15,757	0 (0.0)	0 (0.0)	0 (0.0)	0 (0.0)
Bellarine and Surf Coast endemic SA2					
Ocean Grove–Barwon Heads	29,382	45 (**76.8**)	28 (**41.0**)	37 (**46.2**)	47 (**51.0**)
Point Lonsdale–Queenscliff	4,746	38 (**303.3**)	20 (**157.9**)	7 (**53.0**)	21 (**144.3**)
Portarlington	8,541	0 (0.0)	5 (**22.8)**	4 (**16.8**)	4 (**15.2**)
Lorne–Angelsea‡	5,615	0 (0.0)	0 (0.0)	6 (**37.1**)	8 (**46.8**)

*Data presented are for regions of interest in SA2 of Geelong and other recognized Bellarine Peninsula and Surf Coast disease-endemic areas. Bold text indicates incidence >10 per 100,000 person-years. SA2, Australian Bureau of Statistics Statistical Area 2. †Australian Bureau of Statistics estimated resident population for 2020. ‡Includes Aireys Inlet, Angelsea, Lorne. Aireys Inlet, 7 cases since 2017; Anglesea, 5 cases since 2017.

Of the 49 Buruli ulcer cases diagnosed since 2020 in Geelong suburbs, 39 case-patients resided within areas included in the 2020 possum excreta survey (Figure 2). The median distance from case-patient residence to *M. ulcerans*–positive possum feces was 283 meters (Table 2). In all, 16/39 (41%) of the cases resided within 200 meters of a positive possum sample. The median distance to a positive possum sample was lower for cases within the identified clusters in Belmont, Highton, and Newtown (199 meters; 50% of case-patients resided within 200 meters of a positive possum sample) than cases who were not within the identified clusters (median 1,177 meters; 23% within 200 meters; p = 0.040 by rank-sum test). For cases within the central areas sampled in both 2020 and 2022 fecal surveys, the median distance from a case residence to a prior positive possum sample was only 108 meters (interquartile range [IQR] 82–263 meters).

**Table 2:** Proximity and timing of human cases of Buruli ulcer in areas with *Mycobacterium ulcerans–*positive possum fecal samples collected in 2020 and 2022, Australia*

Characteristic	Cases diagnosed since 2020	Cases within detected clusters	Cases not in detected clusters	Cases in 2-year follow-up survey area
Total cases	39	26	13	24
Distance from positive possum fecal sample, meters				
Median (IQR)	283 (92–1,025)	199 (88–393)	1177 (209–1,256)	108 (82–263)
Range	35–1,867	35–1,531	70–1,867	35–405
Proximity				
<200 meters	16 (41)	13 (50)	3 (23)	16 (67)
<500 meters	26 (67)	20 (77)	6 (46)	24 (100)
Months between possum detections and cases, median (IQR)	17 (8–29)	19 (8–31)	16 (11–26)	12 (7–28)

*Values are no. (%) except as indicated. Clusters here include the 3 identified clusters (Belmont, Highton, Newtown, as presented in Figure 2, panel C). All fecal samples positive by IS2404 from any of the surveys (presurvey samples September 2019, main 2020 survey, 3-mo, 6-mo or 24-mo, follow-up) were included in the measurement of proximity; the closest sample contributing to these summary measures. †Month calculated as days divided by 30. Two cases with closest proximity samples <100 meters in 2022 (postdiagnosis in 2021) have been excluded from this time calculation, although both had relatively proximal detections (<250 meters) before diagnosis also.

The most geographically proximal detections of *M. ulcerans* in possum fecal samples preceded the emergence of human Buruli ulcer cases by up to 39 (IQR 8−29) months (Table 2). For cases in identified clusters, the most geographically proximal positive possum fecal samples to cases occurred with an IQR of 7–31 months before the case diagnosis, apart from 2 cases in which the closest positive detection (<100 meters) occurred after case diagnosis. For those cases, other positive possum feces within 250 meters of the case residence were detected 16 and 18 months before the case diagnosis. Of note, we observed few Buruli ulcer cases in the northern suburbs of Geelong during 2011–2022. Possum population density was lower in these suburbs; few to no possum feces were present during the 2020 excreta survey (Appendix Figure 1). No cases were reported in North Geelong-Bell Park SA2; 1 of the 2 cases reported in 2018 in Corio-Norlane SA2 resided within 344 meters of the subsequent single *M. ulcerans*–positive possum fecal detection in 2020. Analysis examining the timeliness of diagnosis in BSW demonstrated delays in Buruli ulcer diagnosis in emerging endemic areas.

## Conclusions

Buruli ulcer endemicity in Victoria, Australia, is evolving. Our multidisciplinary investigation of human Buruli ulcer cases and *M. ulcerans* shedding in local possums describes the speed and pattern of spread in a large noncoastal urban center in Victoria. Since 2017, Buruli ulcer cases have increased and clustered within 3 Geelong suburbs, predominantly near possums shedding *M. ulcerans* in feces. Possum detection precedes human cases by months to years, suggesting that possum fecal surveillance for *M. ulcerans* may be useful in identifying emerging areas before the onset of human cases. Our findings support a recently published statistical model to predict the location of future human Buruli ulcer cases based on previous human cases and the location of positive possum fecal specimens (*10*); here, we defined the temporal relationship whereby infection appears in possums before humans in an emerging disease area. Furthermore, we quantified the time between the positive detections in possum populations and subsequent human cases. Our results suggest that possum fecal positivity may provide an early warning signal of emerging at-risk areas for Buruli ulcer. In Geelong, the Barwon South West Public Health Unit, established in late 2020, uses this information (*13*) to inform targeted efforts to enhance community awareness and clinician knowledge of the condition in relation to recommended behaviors aimed at disease prevention (*7*) and for testing for more timely diagnosis (*14*). Evidence that such recommendations reduce Buruli ulcer incidence is limited; further research is required to investigate this possibility. As human cases emerge, *M. ulcerans* detections in possum feces may help delineate new areas of local transmission from travel-related acquisition within known endemic areas, assisted by research into the ecology and transmission pathways of Buruli ulcer to develop interventions to reduce disease in both human and wildlife populations. Early detection of *M. ulcerans* shedding in possums may inform decisions on target areas to implement and evaluate any such potential future interventions in Victoria.

This article was preprinted at https://medrxiv.org/cgi/content/short/2024.05.03.24306731v1.

Appendix Additional information about *Mycobacterium ulcerans* found in possum feces as an indicator of emergence of Buruli ulcer in humans, Australia.

## References

[1] World Health Organization. Working to overcome the global impact of neglected tropical diseases: first WHO report on neglected tropical diseases. Geneva: The Organization; 2010.

[2] Merritt RW, Walker ED, Small PL, Wallace JR, Johnson PD, Benbow ME, et al. Ecology and transmission of Buruli ulcer disease: a systematic review. PLoS Negl Trop Dis. 2010;4:e911. 10.1371/journal.pntd.0000911 21179505 PMC3001905

[3] O’Brien DP, Jeanne I, Blasdell K, Avumegah M, Athan E. The changing epidemiology worldwide of *Mycobacterium ulcerans.* Epidemiol Infect. 2018;147:e19. 10.1017/S0950268818002662 30293536 PMC6518549

[4] Loftus MJ, Tay EL, Globan M, Lavender CJ, Crouch SR, Johnson PDR, et al. Epidemiology of Buruli ulcer infections, Victoria, Australia, 2011–2016. Emerg Infect Dis. 2018;24:1988–97. 10.3201/eid2411.171593 30334704 PMC6199991

[5] Buultjens AH, Vandelannoote K, Meehan CJ, Eddyani M, de Jong BC, Fyfe JAM, et al. Comparative genomics shows that *Mycobacterium ulcerans* migration and expansion preceded the rise of Buruli ulcer in southeastern Australia. Appl Environ Microbiol. 2018;84:e02612–7. 10.1128/AEM.02612-17 29439984 PMC5881063

[6] Mee PT, Buultjens AH, Oliver J, Brown K, Crowder JC, Porter JL, et al. Mosquitoes provide a transmission route between possums and humans for Buruli ulcer in southeastern Australia. Nat Microbiol. 2024;9:377–89. 10.1038/s41564-023-01553-1 38263454 PMC10847040

[7] McNamara BJ, Blasdell KR, Yerramilli A, Smith IL, Clayton SL, Dunn M, et al. Comprehensive case-control study of protective and risk factors for Buruli ulcer, southeastern Australia. Emerg Infect Dis. 2023;29:2032–43. 10.3201/eid2910.230011 37735741 PMC10521623

[8] Fyfe JA, Lavender CJ, Handasyde KA, Legione AR, O’Brien CR, Stinear TP, et al. A major role for mammals in the ecology of *Mycobacterium ulcerans.* PLoS Negl Trop Dis. 2010;4:e791. 10.1371/journal.pntd.0000791 20706592 PMC2919402

[9] Blasdell KR, McNamara B, O’Brien DP, Tachedjian M, Boyd V, Dunn M, et al. Environmental risk factors associated with the presence of *Mycobacterium ulcerans* in Victoria, Australia. PLoS One. 2022;17:e0274627. 10.1371/journal.pone.0274627 36099259 PMC9469944

[10] Vandelannoote K, Buultjens AH, Porter JL, Velink A, Wallace JR, Blasdell KR, et al. Statistical modeling based on structured surveys of Australian native possum excreta harboring *Mycobacterium ulcerans* predicts Buruli ulcer occurrence in humans. eLife. 2023;12:e84983. 10.7554/eLife.84983 37057888 PMC10154024

[11] Australian Bureau of Statistics. Regional population, estimated residential population 2001–2020. Canberra: The Bureau; 2022.

[12] Blasdell KR, McNamara B, O’Brien DP, Tachedjian M, Boyd V, Dunn M, et al. Environmental risk factors associated with the presence of *Mycobacterium ulcerans* in Victoria, Australia. PLoS One. 2022;17:e0274627. 10.1371/journal.pone.0274627 36099259 PMC9469944

[13] Department of Health Victoria. Local public health units. 2023 [cited 2024 Feb 5]. https://www.health.vic.gov.au/local-public-health-units

[14] Coutts SP, Lau CL, Field EJ, Loftus MJ, Tay EL. Delays in patient presentation and diagnosis for Buruli ulcer (*Mycobacterium ulcerans* infection) in Victoria, Australia, 2011–2017. Trop Med Infect Dis. 2019;4:100. 10.3390/tropicalmed4030100 31277453 PMC6789443

