# Supplementary material for: Mycobacterium ulcerans in Possum Feces before Emergence in Humans, Australia
Source: Emerg Infect Dis. 2025 Mar;31(3):569–73. doi: 10.3201/eid3103.240657 (PMC11878303; doi:10.3201/eid3103.240657)
Supplement: Appendix — Additional information about Mycobacterium ulcerans found in possum feces as an indicator of emergence of Buruli ulcer in humans, Australia. [file 24-0657-Techapp-s1.pdf]

Article DOI: https://doi.org/10.3201/eid3103.240657

EID cannot ensure accessibility for supplementary materials supplied by authors. Readers who have difficulty accessing supplementary content should contact the authors for assistance.

Mycobacterium ulcerans in Possum Feces before Emergence in Humans, Australia

Appendix

Supplementary Methods

Case Definition and Data Sources

This study included laboratory-confirmed and probable cases of BU, as previously described (*1*). We extracted data on all BU cases notified to the Department of Health (DH) Victoria between 1 January 2011 and 31 December 2022, from the Public Health Events Surveillance System (PHESS) for cases with a residential address within the Barwon South West (BSW) Public Health Unit catchment in southwest Victoria. Cases were geo-coded to longitude and latitude and to the corresponding Australian Bureau of Statistics ABS 2016 Mesh Block geographic statistical area (*2*). Mesh blocks are the smallest unit of statistical geography in the ABS Australian Statistical Geography Standard (ASGS), in this instance denoting areas with 4–84 persons and 3–40 dwellings. These were then aggregated for analysis to broader geographic areas within the ASGS, namely Statistical Area 1 (SA1) and Statistical Area 2 (SA2) (*2*). SA2 allow descriptions that equate loosely to known suburbs while having accurate GIS mapping boundaries and derivation of population estimates over time. The following central Geelong locations (SA2) and 2020 estimated residential populations were the focus of our study: Belmont (14,829 residents), Corio-Norlane (27,622), Geelong (13,781), Geelong West-Hamlyn Heights (21,272), Grovedale (31,579), Highton (23,869), Newcomb-Moolap (15,089), Newtown (10,945) and North Geelong-Bell Park (15,757) (*3*) (Appendix Figure 1). Known proximal endemic areas for comparison included the following SA2: Ocean Grove - Barwon Heads, Point Lonsdale -

Queenscliff, and Portarlington on the Bellarine Peninsula and Lorne-Anglesea on the Surf Coast (Figure 1).

Epidemiology of Cases

We used descriptive statistics to summarize age, sex, size of the BU lesion(s), season of symptom onset, time between symptom onset and diagnosis (based on case notification date), time between symptom onset and first healthcare presentation (*presentation delay*), and time between first healthcare presentation to diagnosis (*diagnosis delay*). For proportions, we calculated 95% confidence intervals using the binomial distribution. We examined reported travel to other endemic areas (as reported by treating clinicians and/or cases) and classified this to determine- those with either single or multiple visits to proximal (Bellarine or Surf Coast areas) or more distant known endemic regions of Victoria and those without. Differences in these characteristics across time periods (2011–2019, 2020–2022) and presentation and diagnosis delays were assessed using Chi-square tests or tests for difference of median values.

Geographic Distribution of BU Cases over Time

We examined aggregate case numbers and incidence per year at SA2 for the central Geelong suburbs and known endemic areas of the Bellarine and Surf Coast. Case numbers and case incidence at Statistical Area 1 (SA1) in central Geelong were mapped to visualize local clustering and the spread of cases, and changes in case incidence (total BU cases per 100,000 population) over time, across four time periods: 2011–2013, 2014–2016, 2017–2019, 2020–2022. For these analyses, the resident population within each SA1 and SA2 was sourced from ABS Estimated Resident Population (ERP) for the areas of interest from 2011 to 2020 (*3*). Population estimates for 2021 and 2022, which were not available for these geographic boundaries at the time of analysis, were derived from the average population growth estimated by the ERP of the previous 5 years.

Spatiotemporal Analysis of Clustering of Human BU Cases

We examined geographic clustering of cases in central Geelong suburbs in a space-time analysis that examined clustering of cases mapped to Mesh blocks over time. We used Poisson models in SatScan (trademark of Martin Kulldorff; http://www.satscan.org) (*4*) to scan across the geographic locations over time to detect areas and time periods where the number of cases significantly exceeded the expected number of cases for the population to test the null hypothesis

that cases are equally geographically distributed across the region for the period. For each significant cluster, we have reported the observed number of cases, the relative risk compared to the expected number under the null hypothesis and level of statistical significance. We conducted a subsequent, additional SatScan spatiotemporal analysis that allowed partial overlapping clusters (cluster centers were not within other clusters) and with the time-period restricted to single year intervals to further explore how the spread of the tightly clustered human cases within the Belmont area had emerged.

Timing and Proximity of BU Cases to Possum Fecal Samples Positive for *M. ulcerans* DNA

We used data from a systematic possum fecal survey conducted in Geelong suburbs in 2020 (*5*), as well as follow-up surveys that were conducted in a smaller subset of Geelong regions at three, six and 24 months after the initial survey (Appendix Figure 1), and several samples collected in the central Belmont area on 26 September 2019, before the main survey. Details of survey collection methods have been published previously (*5*). In brief, samples were collected across grid coordinates located 200m apart. Samples were collected from the nearest publicly accessible location to the grid coordinate (e.g., nature strips, footpaths). Field researchers also noted the coordinates and the date where no samples were found. Collected samples were homogenized, extracted, and analyzed by IS2404 PCR as described previously (*6*), to detect *M. ulcerans* DNA.

We calculated the proximity of human cases notified in 2020–2022 to possum fecal samples positive for *M. ulcerans* DNA. We have reported the median and interquartile range of the distance between the most proximal positive possum samples and residences of BU cases, as well as the number and proportion of cases within varying distance radius (200 m and 500 m). This analysis of geographic proximity was undertaken for all cases residing in areas of the 2020 possum survey, for cases in the identified clusters, and for cases residing within areas with possum fecal testing in 2022. We also calculated the timing of case diagnosis in relation to the most proximal fecal detection in months.

Supplementary Results

Epidemiology of BU Cases

Incidence of BU cases in four time periods (2011–2013, 2014–2016, 2017–2019, 2020–2022) are shown in Appendix Figure 2, and BU cases number in emerging and established endemic areas in Appendix Figure 3A-B. Demographics, epidemiology, and clinical characteristics of cases within the Geelong areas of interest in the period 2011–2019 compared to those notified in 2020–2022 are presented in Appendix Table 1. The age of cases did not differ between the two time periods; however the proportion of female cases were higher (not statistically significant) in the period 2020–2022 compared to 2011–2019 ($p = 0.052$). Winter was the main season of symptom onset in both time periods. The proportion of cases without travel to other endemic areas was higher, but not statistically significant, in the period 2020–2022 (42%; 95% CI 32, 51) compared to 2011–2019 (29%; 95% CI 20, 37). Of those reporting travel, all cases reported visiting endemic areas on the Bellarine Peninsula or Surf Coast; additionally, 36% of these also reported travel to other endemic areas in Victoria. Time from symptom onset to diagnosis was similar in 2011–2019 and 2020–2022 (median 7–8 weeks, Appendix Table 1), but greater than that in other previously recognized endemic areas of the BSW region (Appendix Table 2 and Appendix Figure 4). The duration from symptoms to first seeking healthcare was similar in both the Geelong suburbs of interest and known endemic areas (median and IQR: 2.9 (0.9, 6.0) and 3.0 (1.0, 5.8) weeks, $p = 0.63$), while the time from healthcare presentation to diagnosis differed; median and IQR of 2.6 (1.0, 5.9) weeks compared to 1.0 (0.6, 2.7) weeks in prior endemic areas, $p = 0.002$ (Appendix Table 2, Appendix Figure 4), suggesting a delay in diagnosis.

Distribution of Human BU cases over Time and Proximity to Possum Fecal Samples Positive for *M. ulcerans* DNA

Spatiotemporal analyses identified three clusters suggesting local transmission in Geelong suburbs; Belmont (a cluster of 22 cases in 2019–2022), Highton (11 cases in 2022) and Newtown (3 cases in 2020–2021), with case numbers substantially higher than would be expected if cases followed a random geographic distribution (Appendix Figure 5). Additional spatiotemporal analysis with overlapping clusters and single year periods confirmed the direction spread of BU cases from the initial Belmont cluster (Appendix Figure 6), this also reflected the

distribution of positive possum feces in this central cluster observed across the two survey periods (2020 and 2022, Figure 2D in main manuscript). Appendix Figure 5 also highlights the BU case clusters in relation to rivers and parks and reserves which surround the Belmont area to the North, the West and South, while Appendix Figure 7 shows examples of the vegetation and powerlines that may facilitate possum populations and mobility in these emerging areas.

References

1. Betts JM, Tay EL, Johnson PDR, Lavender CJ, Gibney KB, O'Brien DP, et al. Buruli ulcer: a new case definition for Victoria. Commun Dis Intell. 2018;2020:44-50. https://doi.org/10.33321/cdi.2020.44.93

2. Australian Bureau of Statistics. 1270.0.55.001. Australian Statistical Geography Standard (ASGS): volume 1—main structure and greater capital city statistical areas, July 2016. Canberra: Australian Bureau of Statistics; 2017.

3. Australian Bureau of Statistics. Regional population, estimated residential population 2001–2020. Canberra: Australian Bureau of Statistics; 2022.

4. Kulldorff M. A spatial scan statistic. Commun Stat Theory Methods. 1997;26:1481–96. https://doi.org/10.1080/03610929708831995

5. Vandelannoote K, Buultjens AH, Porter JL, Velink A, Wallace JR, Blasdell KR, et al. Statistical modeling based on structured surveys of Australian native possum excreta harboring *Mycobacterium ulcerans* predicts Buruli ulcer occurrence in humans. eLife. 2023;12: e84983. PubMed https://doi.org/10.7554/eLife.84983

6. Blasdell KR, McNamara B, O'Brien DP, Tachedjian M, Boyd V, Dunn M, et al. Environmental risk factors associated with the presence of *Mycobacterium ulcerans* in Victoria, Australia. PLoS One. 2022;17:e0274627. PubMed https://doi.org/10.1371/journal.pone.0274627

7. World Health Organization. Treatment of *Mycobacterium ulcerans* disease (Buruli ulcer): guidance for health workers. Geneva: The Organization; 2012.

Appendix Table 1. Epidemiologic and clinical characteristics of Buruli ulcer cases in Geelong suburbs during each of the time periods and overall

	2011–2019, N = 31		2020–2022, N = 49		Total, N = 80		
Characteristic	n	% (95%CI)	n	% (95%CI)	n	% (95%CI)	p value†
Age, y							0.95
0–19	4	13 (6–19)	6	12 (6–19)	10	13 (6–19)	
20–39	9	29 (20–38)	12	24 (16–33)	21	26 (18–35)	
40–59	9	29 (20–38)	14	29 (20–37)	23	29 (20–38)	
60+	9	29 (20–38)	17	35 (25–44)	26	33 (23–42)	
Median (IQR)		40 (29–61)		44 (31–65)		42 (29–63)	
Sex							0.05
Female	10	32 (23–41)	28	57 (47–67)	38	48 (38–57)	
Male	21	68 (59–77)	21	43 (33–53)	42	53 (43–62)	
Other	0		0		0		
Season of symptom onset*							0.47
Summer	4	13 (6–19)	4	8 (3–14)	8	10 (4–16)	
Autumn	8	26 (17–34)	7	14 (7–21)	15	19 (11–26)	
Winter	13	42 (32–52)	27	55 (45–65)	40	50 (40–60)	
Spring	6	19 (12–27)	11	22 (14–31)	17	21 (13–29)	
Time from symptom onset* to notification (weeks)							0.96
Median (IQR)	21	7.0 (5.7–8.4)	44	7.6 (4.1–13.0)	65	7.4 (4.9–12.3)	
Lesion size: WHO classification							0.61
Category 1	19	61 (52–71)	34	69 (60–78)	53	66 (57–76)	
Category 2	4	13 (6–19)	5	10 (4–16)	9	11 (5–17)	
Category 3	1	3 (0,7)	5	10 (4–16)	6	8 (2–13)	
Missing	7	23 (14–31)	5	10 (4–16)	12	15 (8–22)	
Travel to endemic regions outside area of residence							
No travel	8	29 (20–37)	20	42 (32–51)	28	37 (27–46)	0.25
Travel to endemic areas outside Geelong suburbs	20	71 (63–80)	28	58 (49–68)	48	63 (54–73)	
Missing exposure data	3		1		4		

*Missing first symptom onset (pre 2020, n = 10; 2020–2022 n = 5), these individuals were excluded from time from symptoms to notification, season of notification was used instead for season of symptom onset. World Health Organization (WHO) lesion severity classification (7): Category 1, single lesion <5 cm diameter; Category 2, single lesion 5–15 cm diameter; Category 3, a single lesion >15cm diameter, multiple lesions, lesions at a critical site (e.g., eye) or osteomyelitis.

†$\chi 2$ for differences in the case characteristic across the two time periods on cases with data, those with missing excluded.

Appendix Table 2. Timing of diagnosis after symptom onset, and the presentation delay, and diagnosis delay for Buruli ulcer cases in central Geelong suburbs notified 2011–2022 compared to cases in previously known endemic areas of the Region of Victoria, Australia*

	Geelong suburbs of interest (new areas)		Known endemic areas in BSW†		
Characteristic	N cases	Weeks (median, IQR)	N cases	Weeks (median, IQR)	p value)‡
Overall 2011–2022					
Time from symptoms to diagnosis	65	7.4 (4.9–12.3)	189	5.1 (3.0–9.6)	0.002
Presentation delay	60	3.0 (1.0–6.1)	181	3.0 (1.0–5.9)	0.60
Diagnosis delay	59	2.7 (1.1–4.9)	195	0.9 (0.6–2.9)	<0.0001
2011–2019					
Time from symptoms to diagnosis	21	7.0 (5.7–8.4)	133	5.1 (3.0–9.3)	0.03
Presentation delay	18	2.9 (0.9–6.0)	127	3.0 (1.0–5.8)	0.63
Diagnosis delay	21	2.9 (0.9–4.4)	138	1.0 (0.6–2.9)	0.03
2020–2022					
Time from symptoms to diagnosis	44	7.6 (4.1–13.0)	56	4.9 (3.4–9.7)	0.06
Presentation delay	42	3.0 (1.0–6.3)	54	2.3 (1.0–6.0)	0.66
Diagnosis delay	38	2.6 (1.1–5.9)	57	1.0 (0.6–2.7)	0.002

*Presentation delay is time from symptom onset to first healthcare presentation. Diagnosis delay is time from healthcare presentation to diagnosis. Cases were excluded if symptom onset date–date of first presentation were missing or invalid. BSW, Barwon South West; IQR, interquartile range.
†Known endemic areas included SA2 of "Ocean Grove - Barwon Heads," "Portarlington," "Point Lonsdale - Queenscliff," "Lorne - Anglesea," "Lorne."
‡Wilcox rank-sum test comparing Geelong suburbs to known endemic areas.

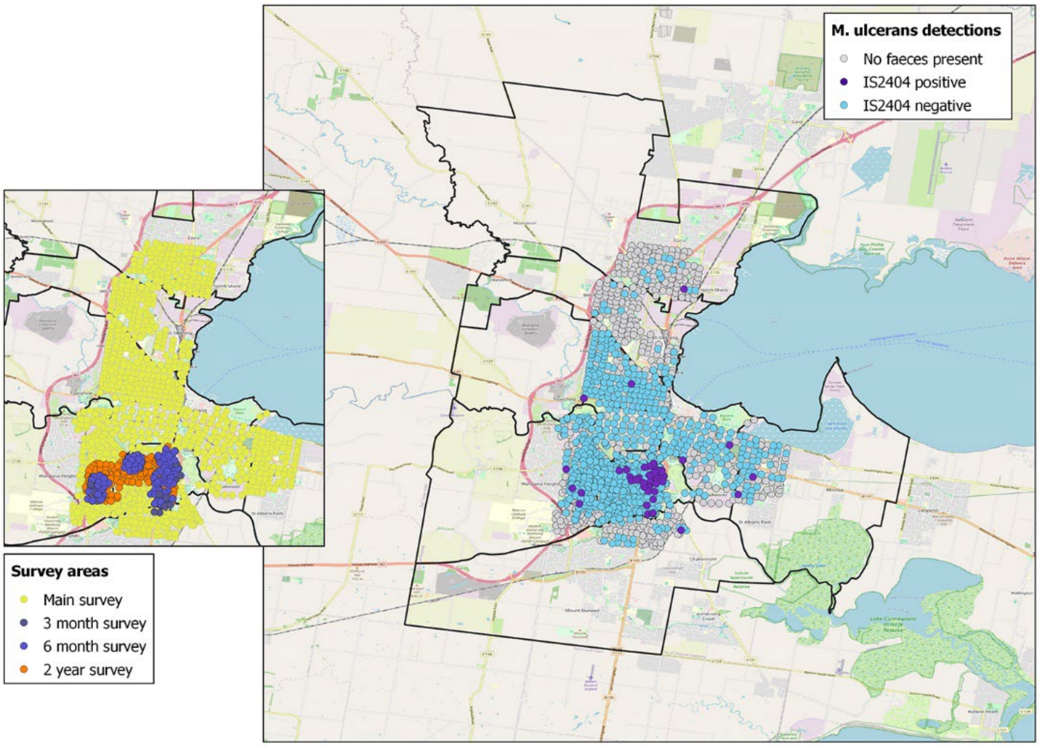

Appendix Figure 1. Map of possum fecal sample collection (inset); the main survey (conducted Jan-April 2020–yellow; 3-month follow-up (May-Jun 2020) and 6-month follow-up (August–September 2020)–purple; areas surveyed in the 24-month follow-up (February to April 2022) in orange. Note 24-month survey areas overlay Main survey areas and 3- and 6- month areas. Map of possum feces locations and *M. ulcerans* detections via IS2404 PCR (main map) – gray points were surveyed but no feces were present.

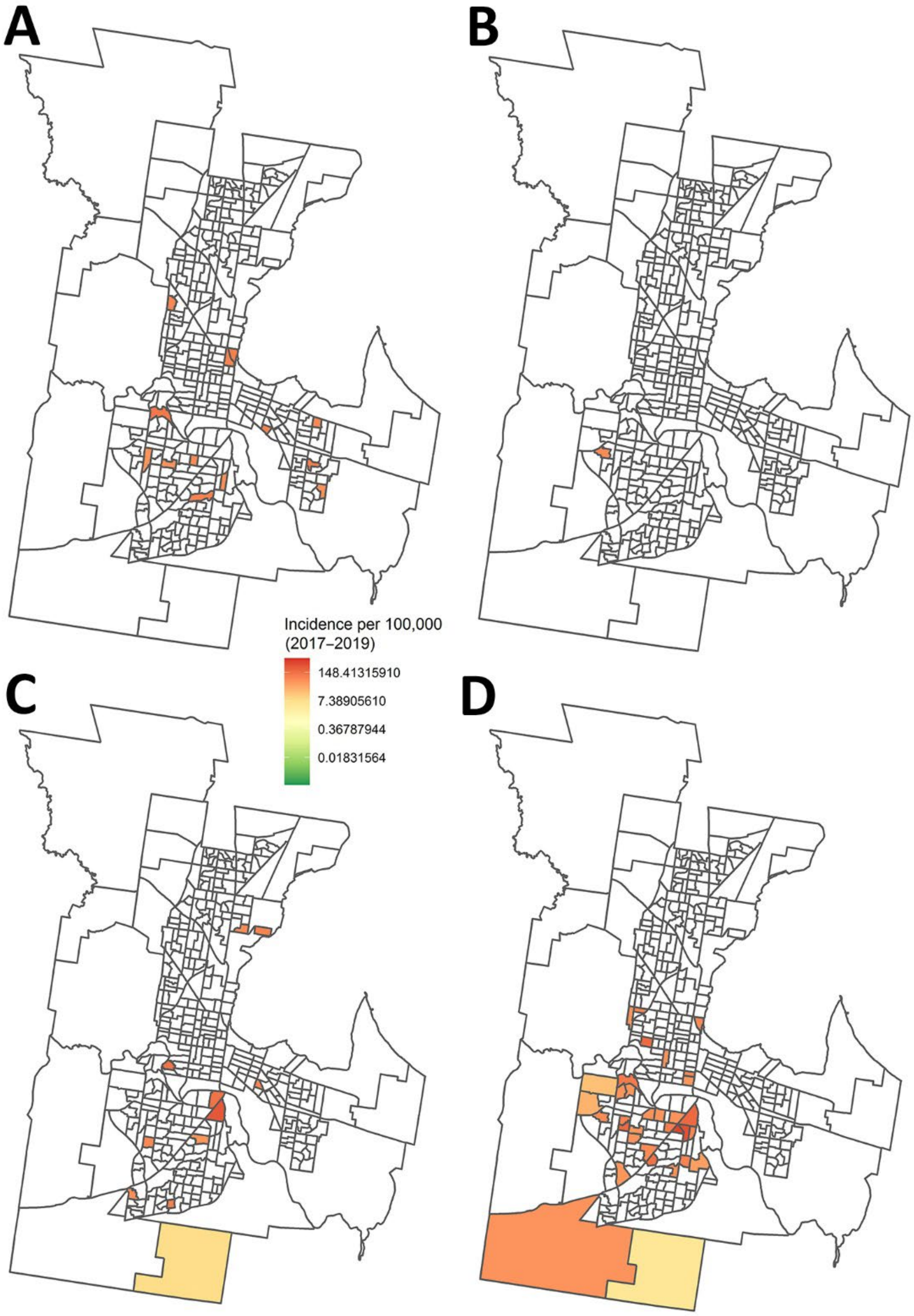

Appendix Figure 2. Geographic distribution of cases per 100,000 person-years (shown on log-scale) across the four time periods (A) 2011–2013–(B) 2014–2016–(C) 2017–2019 and (D) 2020–2022. Note areas with 0 cases shown in white; this also includes n = 16 areas without any estimated residential population (i.e.–parkland).

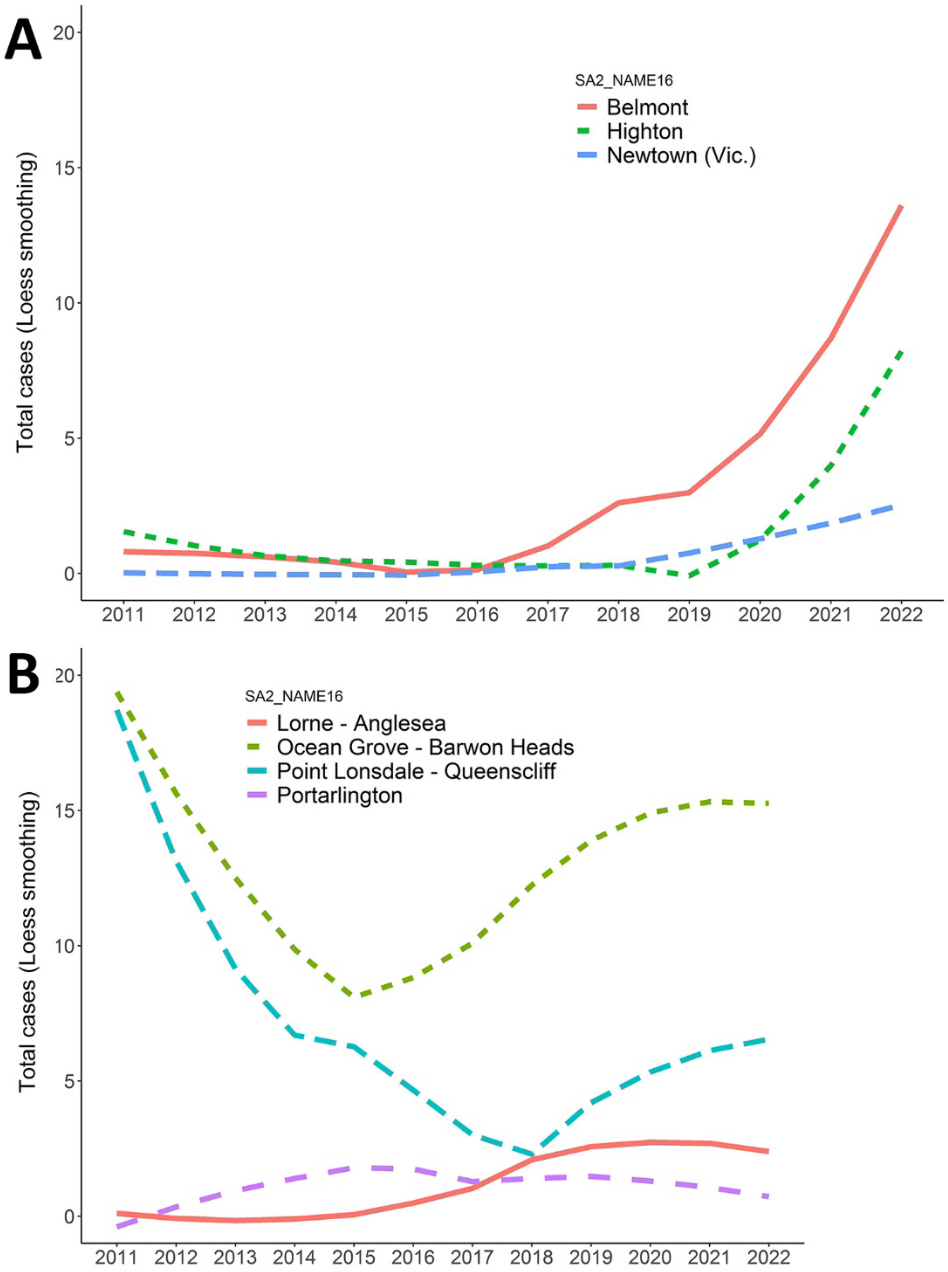

Appendix Figure 3. A) Trends in total BU cases numbers in SA2 with recent increases in transmission in Geelong. Loess smoothing applied. (B) BU case numbers in SA2 previously recognized as endemic areas for BU with high case incidence in Barwon South West region. Loess smoothing applied.

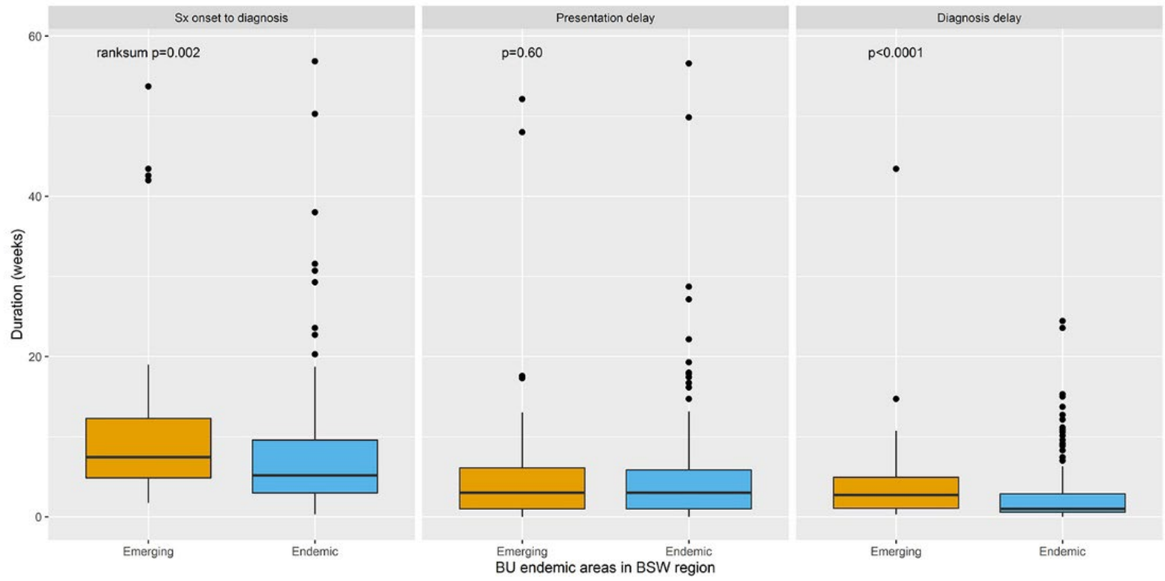

Appendix Figure 4. Time to BU diagnosis after symptom onset and presentation and diagnosis delays for BU cases in central Geelong suburbs from 2011–2022 compared to BU cases in previously known endemic areas of high case incidence in the Barwon South West Region of Victoria–Australia. *Presentation delay* – Time from symptom onset (Sx) to first healthcare presentation for the condition; *Diagnosis delay* – Time from first healthcare presentation to diagnosis. Cases were excluded if symptom onset date–date of first presentation were missing or invalid. P-value of Wilcox rank-sum test comparing median for Geelong suburbs to known endemic areas in BSW (SA2 of Ocean Grove - Barwon Heads–Portarlington–Point Lonsdale - Queenscliff–Lorne - Anglesea–Lorne). Summary data provided in Appendix Table 1.

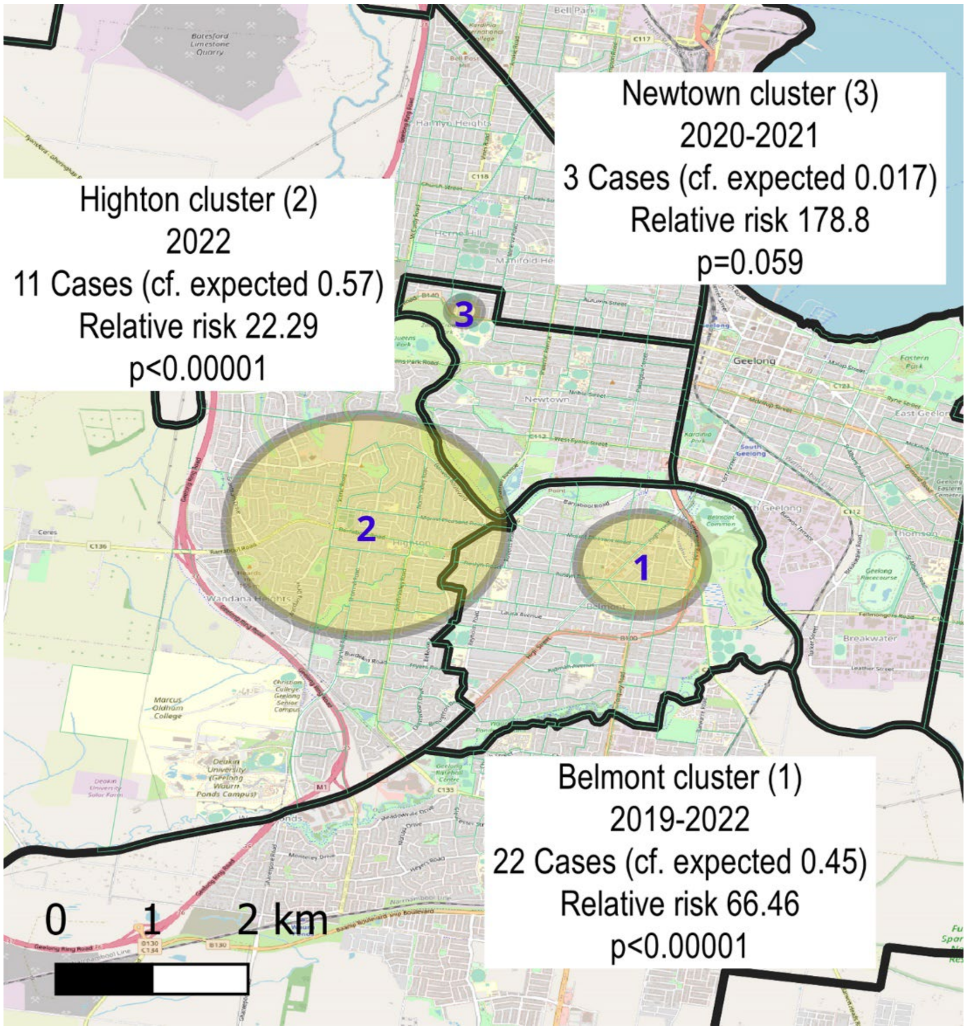

Appendix Figure 5. Map of urban Geelong clusters of BU cases in the period 2011–2022 identified in spatiotemporal analysis (SatScan)–showing the proximity to the Barwon River and Waurn Ponds Creek as well as green space (parks and reserves) and Corio Bay.

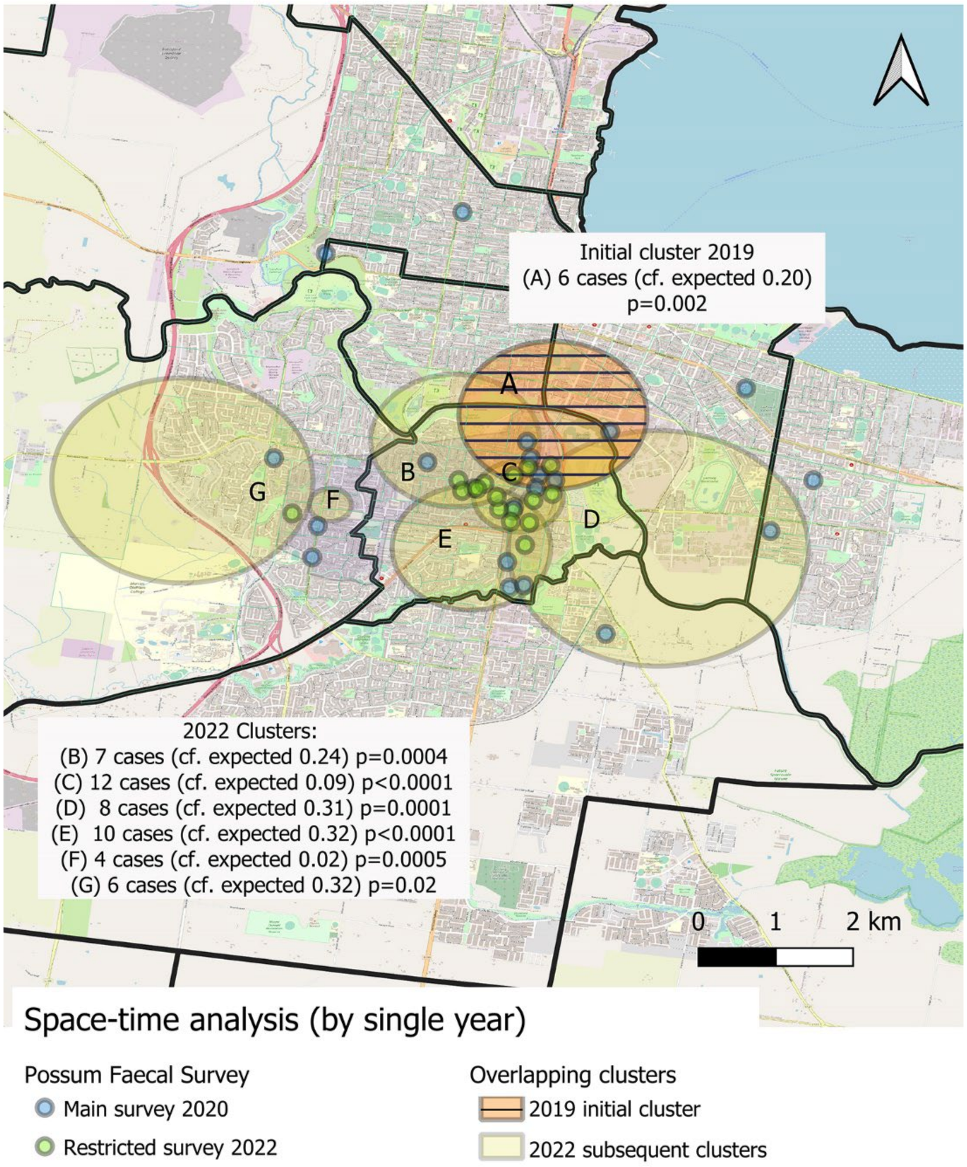

Appendix Figure 6. Spatiotemporal (SatScan) analysis (with overlapping case clusters and single year periods). Outward spread was observed from a central 2019 cluster (Cluster A–orange with striped pattern) with significant surrounding subsequent clusters identified in 2022 (Clusters B–G). The spread and increasing density of *M. ulcerans* in the native possum population is indicated by *M. ulcerans*-positive possum fecal samples from the main 2020 survey (blue) and the restricted 2-year follow-up survey (green).

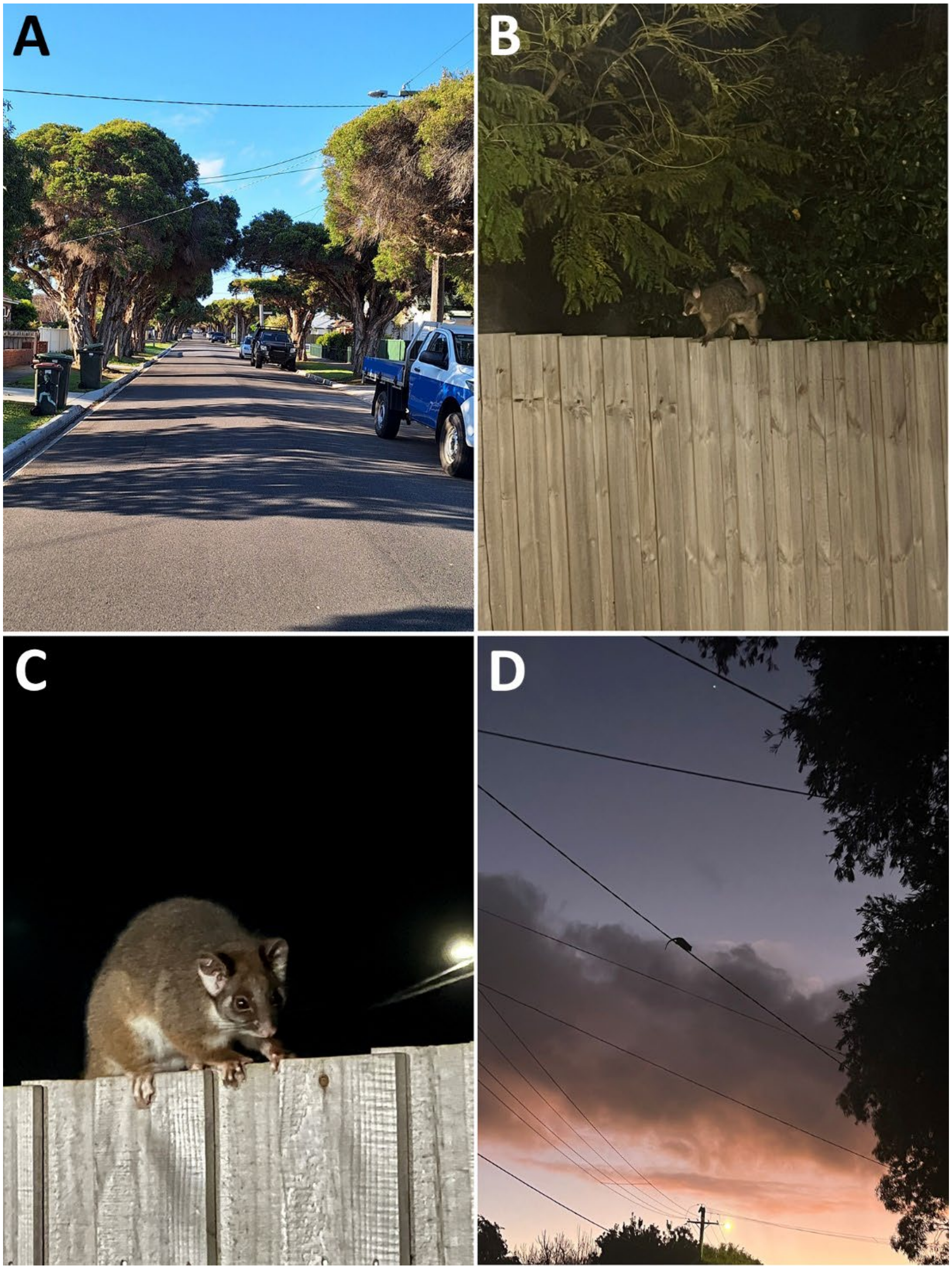

Appendix Figure 7. A) Photograph of typical Belmont street in Geelong showing trees and powerlines that aid in mobility of the local native possum population. B–D) Example photographs of the highly mobile possum population in Highton–City of Geelong.
